# Tissue-Specific Epigenetic Modifications in Root Apical Meristem Cells of *Hordeum vulgare*

**DOI:** 10.1371/journal.pone.0069204

**Published:** 2013-07-31

**Authors:** Agnieszka J. Braszewska-Zalewska, Elzbieta A. Wolny, Lukasz Smialek, Robert Hasterok

**Affiliations:** Department of Plant Anatomy and Cytology, Faculty of Biology and Environmental Protection, University of Silesia, Katowice, Poland; University of Georgia, United States of America

## Abstract

Epigenetic modifications of chromatin structure are essential for many biological processes, including growth and reproduction. Patterns of DNA and histone modifications have recently been widely studied in many plant species, although there is virtually no data on the spatial and temporal distribution of epigenetic markers during plant development. Accordingly, we have used immunostaining techniques to investigate epigenetic modifications in the root apical meristem of *Hordeum vulgare*. Histone H4 acetylation (H4K5ac), histone H3 dimethylation (H3K4me2, H3K9me2) and DNA methylation (5mC) patterns were established for various root meristem tissues. Distinct levels of those modifications were visualised in the root cap, epidermis, cortex and vascular tissues. The lateral root cap cells seem to display the highest level of H3K9me2 and 5mC. In the epidermis, the highest level of 5mC and H3K9me2 was detected in the nuclei from the boundary of the proximal meristem and the elongation zone, while the vascular tissues were characterized by the highest level of H4K5ac. Some of the modified histones were also detectable in the cytoplasm in a highly tissue-specific manner. Immunolocalisation of epigenetic modifications of chromatin carried out in this way, on longitudinal or transverse sections, provides a unique topographic context within the organ, and will provide some answers to the significant biological question of tissue differentiation processes during root development in a monocotyledon plant species.

## Introduction

One of the most intensively studied tissues in higher plants is the root apical meristem (RAM), which contains stem cells [1]–[3]. Recent analyses have indicated the crucial roles of chromatin remodelling in the regulation of stem cell activity [4]–[6], and although many chromatin-remodelling factors have been characterized their molecular mechanisms of actions are still elusive. There is no clear evidence for whether these factors are directly or indirectly involved in the activation and repression of genes during plant development.

**Figure 1:**
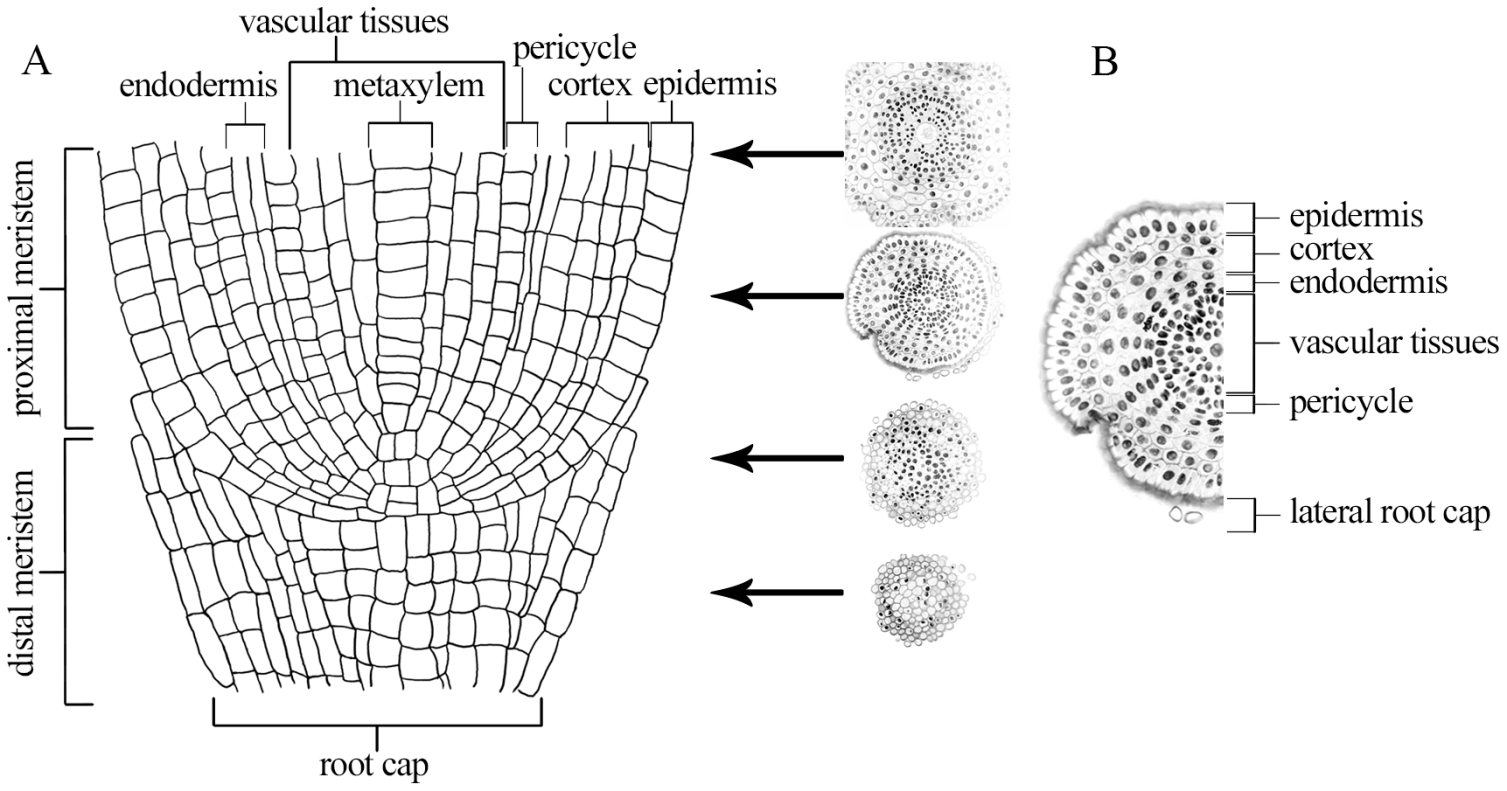
Schematic representation of the *H. vulgare* meristem, longitudinal and transverse sections. **A.** Longitudinal section through the distal and proximal meristem, representative transverse sections from part of the root cap, distal meristem, proximal meristem and boundary between proximal meristem and the elongation zone are marked. **B.** Transverse section across the proximal meristem. Six types of tissues are marked.

Roots develop from the meristematic cells located at the root apex through unknown mechanism(s) of controlled cell proliferation and morphogenesis, which generate radial patterns of tissues within each concentric ring of cells. In a transverse section of a *Hordeum vulgare* meristem five main types of tissues can be distinguished, i.e. a layer of the epidermis, four layers of the cortex, a layer of the endodermis, a layer of the pericycle and vascular tissues (stele) (Figure 1B). The latter has a constant number of eight cells of protophloem to one central cell of the metaxylem. Barley meristems exhibit a closed configuration, where cell boundaries between the cortical epidermis and root cap regions are clearly distinguishable [7]. Root meristem cells show distinct clonal relationships, and both initial cells and their descendants can be easily identified by their position [8]. However, similar to the situation in the shoot stem cells, the fate of a given cell in a root is not permanently fixed, but depends on signals from its neighbours. Laser ablation of individual Quiescent Centre (QC) cells or initials in the *Arabidopsis thaliana* (Arabidopsis) root meristem revealed that these cells can be replaced by their neighbours, which then acquire the appropriate identity [9]–[11]. Although the mechanism underlying this process remains unclear, the correlation between cell position and cell-type differentiation is very well documented during the formation of the root epidermis [12]. For example, Hassan et al. [13] have shown that the fate of Arabidopsis epidermal cells is determined non-cell-autonomously by the action of a zinc finger protein (JACKDAW, JKD) from the underlying cortex cell layer.

Yadav et al. [14] proposed that chromatin in plant stem cells is maintained in a flexible state in order to dynamically balance gene expression. There are several factors that can greatly influence chromatin structure, one of which is modification of histone proteins. Core histones are structurally conserved through evolution and contain flexible N-terminal tails that may be subject to numerous posttranslational covalent modifications, including acetylation, methylation, phosphorylation, ubiquitination, ribosylation, glycosylation, and sumoylation [15]. Acetylated histones are enriched in the regions of chromatin with high DNAse I sensitivity, which correlates with transcriptional activity. Lysine residues at the N-terminal tails of histone proteins are the predominant sites for acetylation (e.g. K9, 14, 18, 23 of H3; K5, 8, 12, 16 of H4) [16], [17]. Histone H3 methylation of lysine K4, K36 and K79 also correlates with active transcription, whereas methylation of K9, K27, and H4K20 are typical hallmarks of silenced chromatin [18]. For example, Arabidopsis heterochromatin has been shown to be associated with a high level of H3K9 dimethylation, whereas its euchromatin is rich in dimethylated H3K4 [19]. Patterns of histone H3 methylation have been studied in plants with small genomes, such as Arabidopsis, which have the majority of their heterochromatin located within their chromocenters [19], [20], as well as in species with larger genomes, e.g. *Hordeum vulgare* and *Vicia faba* [21].

Lysines can be monomethylated (me1), dimethylated (me2), or trimethylated (me3), and each methylation state may have a unique biological function [22], [23]. In *Neurospora* spp., *Saccharomyces pombe* [24], and mammals [25], H3K9me3 is a typical modification for silent chromatin, whereas in plants H3K9me1 and H3K9me2 are enriched in heterochromatin domains, [20] while H3K9me3 is enriched in euchromatin [26]. DNA methylation is often associated with gene silencing and is prominent in heterochromatin. In addition, DNA methylation and histone methylation are intimately linked in plants. For example, mutations in the Arabidopsis *KYP* (histone H3 methyltransferase) gene leads to reductions in DNA methylation at CNG motifs [27].

It is crucial to understand how the regulation of meristematic cell fate and differentiation are co-ordinated to enable root development. Most data on the role of specific genes and transcription factors that control RAM activity and root development comes from Arabidopsis [28]–[30], while studies on other species, such as barley [31], are limited. Despite some immunostaining analyses of histone and DNA modifications in nuclei from roots [16], [19], [21], [32], there is a dearth of comprehensive data on the global levels of these modifications and their tissue-specificity.

To our knowledge this is the first study describing epigenetic modifications visualised in both longitudinal and transverse sections of the root apical meristem, thus enabling a particular focus on their tissue and cell specificity. We have investigated four epigenetic modifications that are linked either to euchromatin (histone H4 acetylation – H4K5ac, and histone H3 methylation – H3K4me2) or heterochromatin (histone H3 methylation – H3K9me2, and DNA methylation – 5mC). Combining immunostaining techniques, we demonstrated spatial distribution of these modifications within the cells of particular RAM tissues.

## Results

Transverse sections included the whole meristem and the beginning of the elongation zone. For clarity of presentation only four representative sections are shown. The transverse sections display the distal meristem or root cap (lateral root cap and columella) and quiescent centre, the proximal meristem and the boundary between the proximal meristem and the elongation zone. The longitudinal sections were excised from the middle part of the meristem, including the whole stele (Figure 1A and 1B).

### Histone H4 acetylation at lysine 5 (H4K5ac)

**Figure 2:**
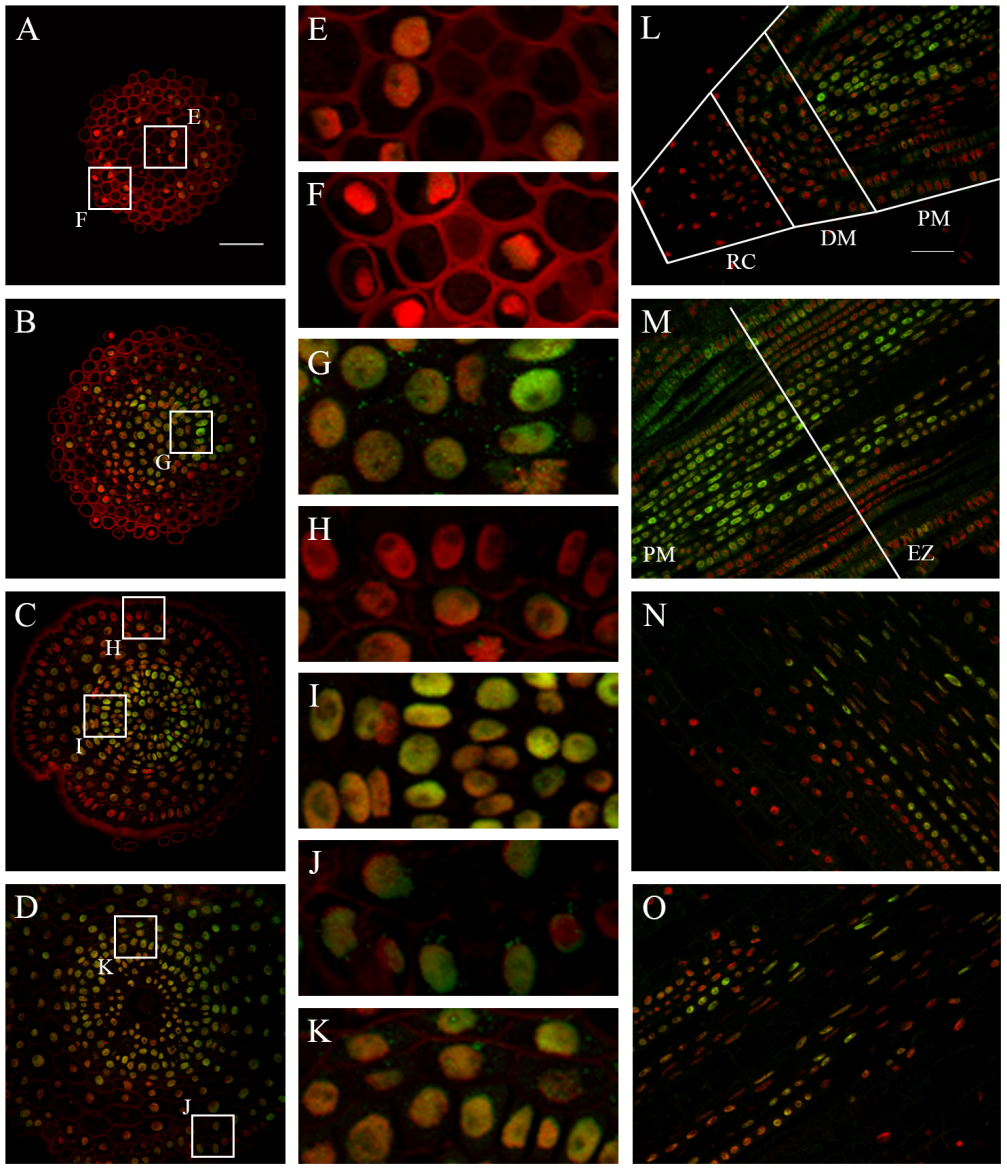
Immunodetection of H4K5ac in the *H. vulgare* root apical meristem. **A–D.** Transverse sections across the root cap (**A**), distal meristem (**B**), proximal meristem (**C**) and the boundary between the proximal meristem and the elongation zone (**D**). **E–K.** Insets show enlargement of the columella root cap cells (**E**), lateral root cap (**F**) vascular cylinder from the distal meristem (**G**), epidermis from the proximal meristem (**H**), endodermis, pericycle and vascular tissues from the proximal meristem (**I**), epidermis from the boundary between the proximal meristem and the elongation zone (**J**), cortex, endodermis and pericycle from the same boundary (**K**). **L–O.** Longitudinal sections through the root cap, distal and proximal meristem (**L**), boundary between the proximal meristem and the elongation zone (**M**), elongation zone (**N**), elongation zone and the beginning of differentiation zone (**O**). RC – root cap, DM – distal meristem, PM – proximal meristem, EZ – elongation zone. Red - DAPI staining (false colour), green - Alexa 488 (immunostaining of H4K5ac). Bars: 50 µm (transverse sections) and 100 µm (longitudinal sections).

The levels of H4K5ac were measured in 1,953 nuclei, which comprised the distal meristem, proximal meristem and the boundary between the proximal meristem and elongation zone. The highest level of this modification in the meristem was detected in vascular tissues and perycycle (Figure 2B, 2C, 2G, 2I, 2L and 2M), contrary to the epidermis (Figure 2C and 2H) and root cap (Figure 2A, 2E, 2F and 2L), which were the lowest. At the boundary between the proximal meristem and elongation zone, H4K5ac was more uniformly distributed over the tissues (Figure 2D, 2J and 2K). At the end of the elongation zone this modification reached the highest levels within the vascular tissues (Figure 2N and 2O, Table 1 and 2).

**Table 1:** Quantitative measurements of the levels of epigenetic modifications.

Mod.	Zone	Low level		Nuclei no.	Medium level		Nuclei no.	High level		Nuclei no.
		DAPI	Alexa 488		DAPI	Alexa 488		DAPI	Alexa 488	
	DM	3.0±1.3	0.1±0.07	78	2.4±1.0	0.3±0.06	331	2.9±1.50	0.9±0.30	58
H4K5ac	PM	3.3±1.7	0.1±0.08	218	2.8±1.6	0.3±0.10	449	3.4±1.60	0.6±0.20	260
	P/E	3.1±1.1	0.2±0.08	308	3.1±1.5	0.3±0.10	251	-	-	-
	DM	1.9±0.8	0.1±0.03	266	3.0±0.7	0.2±0.04	308	4.6±1.20	0.5±0.07	66
H3K9me2	PM	2.0±0.7	0.1±0.02	543	3.6±1.2	0.2±0.08	424	-	-	-
	P/E	2.0±0.9	0.1±0.04	247	-	-	-	3.3±0.90	0.4±0.09	125
	DM	0.6±0.1	0.3±0.09	131	0.9±0.3	1.6±0.50	130	0.6±0.20	2.7±0.90	147
5mC	PM	1.4±0.7	0.1±0.07	867	1.3±0.7	0.8±0.30	752	-	-	-
	P/E	1.2±0.7	0.2±0.08	204	1.0±0.4	0.6±0.10	224	1.0±0.40	1.5±0.70	51
H3K4me2	N/A	N/A	N/A	N/A	N/A	N/A	N/A	N/A	N/A	N/A

Mod. – modification, H4K5ac – histone H4 acetylation at lysine 5, H3K9me2 – histone H3 dimethylation at lysine 9, 5mC – 5-methyl-cytosine, H3K4me2 – histone H3 dimethylation at lysine 4, DM – distal meristem, PM – proximal meristem, P/E - boundary between the proximal meristem and elongation zone, N/A – not analysed. All data are presented in relative units.

**Table 2:** Tissue specificity of the epigenetic modifications under this study.

Mod.	Zone	Low level	Medium level	High level
	DM	Root cap	Cortex, epidermis, vascular tissues	Perycycle
H4K5ac	PM	Epidermis	Cortex	Perycycle, vascular tissues
	P/E	Epidermis, cortex	Vascular tissues	-
	DM	Vascular tissues	Epidermis, cortex	Root cap
H3K9me2	PM	Vascular tissues	Epidermis, cortex	-
	P/E	Cortex	-	Epidermis, vascular tissues
	DM	Epidermis	Cortex	Root cap, vascular tissues
5mC	PM	Epidermis	Cortex, vascular tissues	-
	P/E	Vascular tissues	Cortex	Epidermis
	DM	Epidermis, cortex, vascular tissues	-	Root cap (cytoplasm)
H3K4me2	PM	Epidermis	Cortex	Vascular tissues
	P/E	Epidermis	Cortex, vascular tissues	Protophloem (cytoplasm)

Mod. – modification, H4K5ac – histone H4 acetylation at lysine 5, H3K9me2 – histone H3 dimethylation at lysine 9, 5mC – 5-methyl-cytosine, H3K4me2 – histone H3 dimethylation at lysine 4, DM – distal meristem, PM – proximal meristem, P/E - boundary between the proximal meristem and elongation zone.

### Histone H3 dimethylation at lysine 4 (H3K4me2)

**Figure 3:**
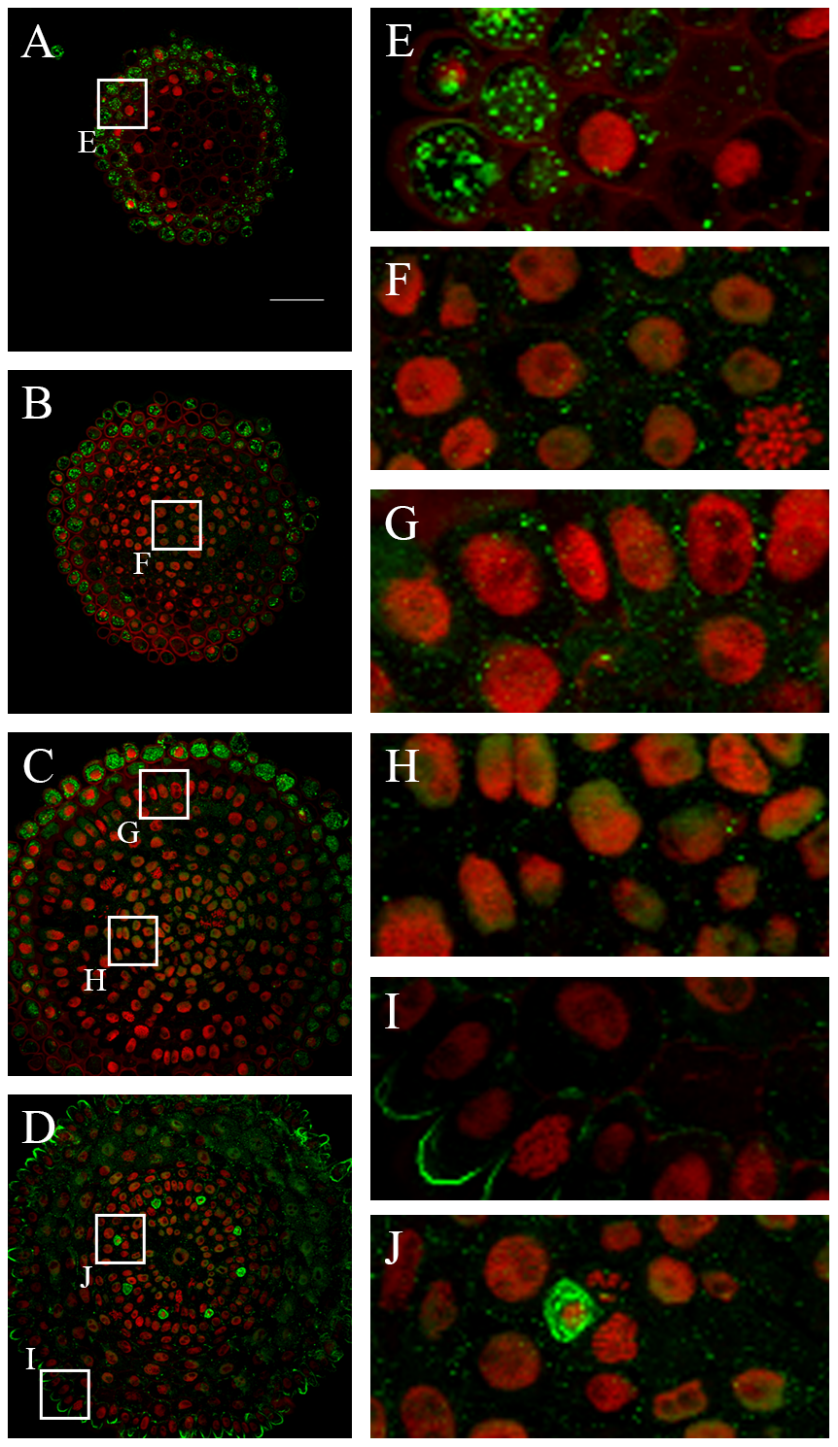
Immunodetection of H3K4me2 in the *H. vulgare* root apical meristem. **A–D.** Transverse sections across the root cap (**A**), distal meristem (**B**) proximal meristem (**C**) boundary between the proximal meristem and the elongation zone (**D**). **E–J.** Insets show enlargement of the root cap cells (**E**), distal meristem (**F**), epidermis and cortex from the proximal meristem (**G**), cortex, endodermis, pericycle and vascular tissues from the proximal meristem (**H**), epidermis from the boundary between the proximal meristem and the elongation zone (**I**) protophloem cell (**J**). Red - DAPI staining (false colour), green - Alexa 488 (immunostaining of H3K4me2). Bar: 50 µm.

The levels of H3K4me2 were quantified based only on a visual interpretation of fluorescence intensity. This was because intensity of the signals of H3K4me2 from the cytoplasm disabled the automatized measurement of signal intensity in the nuclei, using the image cytometer. The most intense signals were detected in the vascular tissues nuclei (Figure 3C and 3H) of the meristem, while the lowest ones were characteristic for the epidermis (Figure 3C, 3G and 3I) and root cap nuclei (Figure 3A and 3B). Strong immunofluorescence was detected in the cytoplasm of the root cap (Figure 3A, 3B and 3E) and protophloem cells (Figure 3D and 3J, Table 2).

### Histone H3 dimethylation at lysine 9 (H3K9me2)

**Figure 4:**
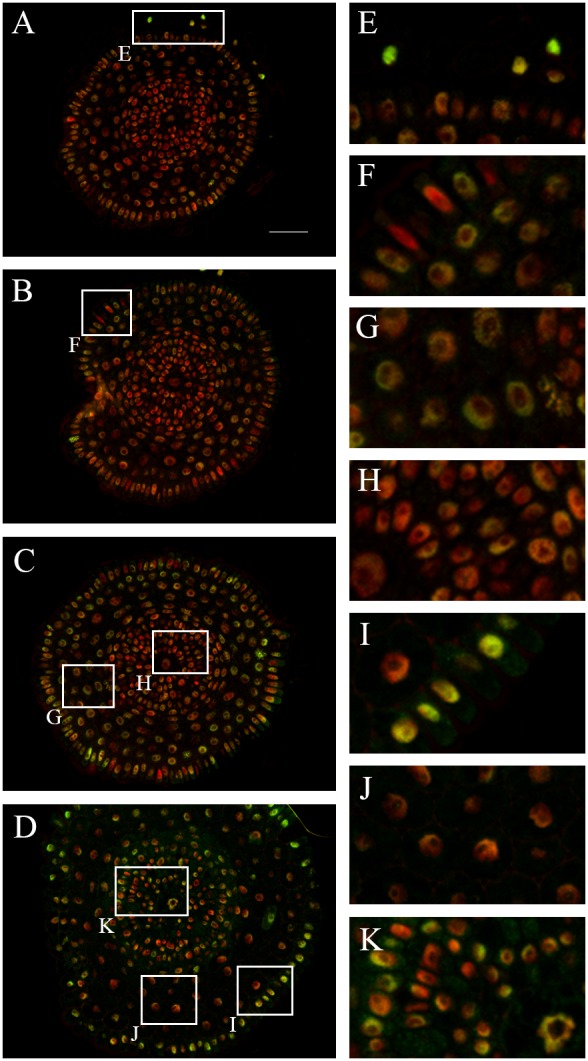
Immunodetection of H3K9me2 in the *H. vulgare* root apical meristem. **A–C.** Transverse sections across the terminal part of the distal meristem (**A**), proximal meristem (**B–C**) and the boundary between the proximal meristem and elongation zone (**D**). **E–K.** Insets show enlargement of the lateral root cap (**E**), epidermis (**F**), cortex (**G**), vascular tissues (**H**) from the proximal meristem; epidermis from the boundary between the proximal meristem and the elongation zone (**I**), cortex (**J**) and vascular tissues from the same zone (**K**). Red - DAPI staining (false colour), green - Alexa 488 (immunostaining of H3K9me2). Bar: 100 µm.

The levels of H3K9me2 were measured in 1,979 nuclei, which comprised the distal meristem, proximal meristem and the boundary between the proximal meristem and the elongation zone. The highest level of this modification was detected in the lateral root cap (Figure 4A and 4E) and epidermis from the boundary between the proximal meristem and elongation zone (Figure 4D and 4I). The vascular tissues from the meristem displayed in general the lowest level (Figure 4B, 4C and 4H), whereas those from the boundary between the proximal meristem and elongation zone (Fig. 4K) had a proportionally high level of this modification. The medium level of H3K9me2 was detected in the cortex nuclei (Figure 4G and 4J, Table 1 and 2).

### 5-methylcytosine (5mC)

**Figure 5:**
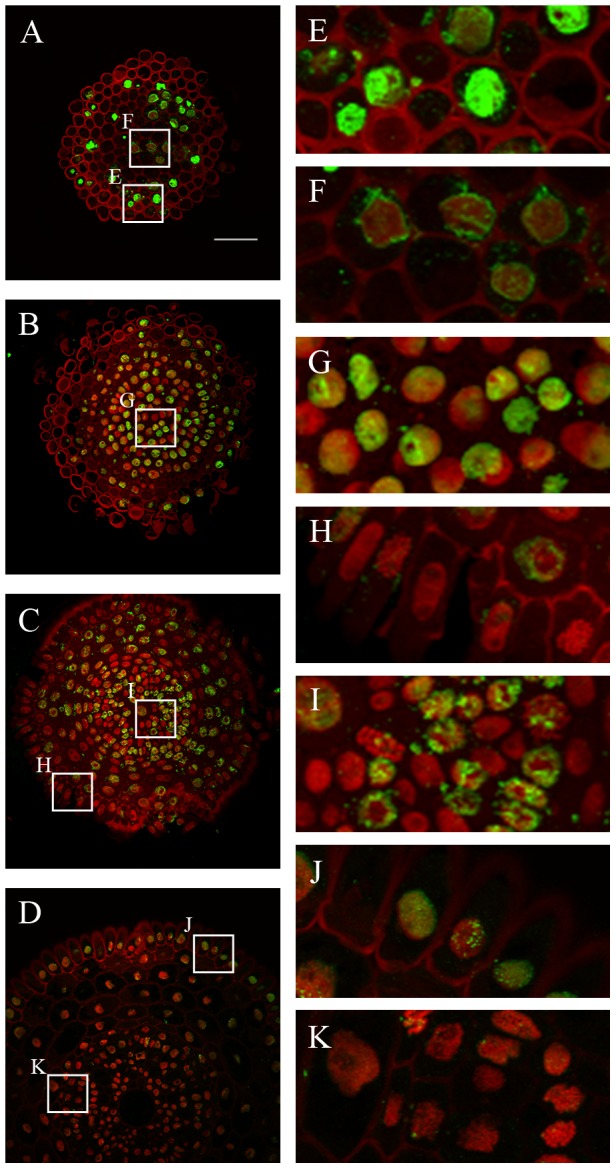
Immunodetection of 5mC in the *H. vulgare* root apical meristem. **A–D.** Transverse sections across the root cap (**A**), distal meristem (**B**), proximal meristem (**C**) and the boundary between proximal meristem and the elongation zone (**D**). **E–K.** Insets show enlargement of the lateral root cap (**E**), columella root cap (**F**), distal meristem (**G**), epidermis and cortex (**H**) from the proximal meristem; endodermis, pericycle and vascular tissues from the proximal meristem (**I**), the epidermis from the boundary between proximal meristem and the elongation zone (**J**), endodermis, pericycle and vascular tissues from the same boundary (**K**). Red - DAPI staining (false colour), green - Alexa 488 (immunostaining of 5mC). Bar: 50 µm.

The levels of 5mC were measured for 2,562 nuclei, which comprised the distal meristem, proximal meristem and the boundary between the proximal meristem and the elongation zone. The highest levels of this modification were detected in the distal meristem (Figure 5A, 5B and 5G), especially in the lateral root cap (Figure 5E). The lowest levels were found in the epidermis of the proximal meristem (Figure 5C and 5H). Interestingly, the epidermis at the boundary between the proximal meristem and elongation zone has the highest level of 5mC of all those tissues (Figure 5D and 5J). The cortex and vascular tissues were characterised by medium levels of this modification (Fig. 5I and 5K, Table 1 and 2).

In conclusion, our results indicate that the levels of modified histones and DNA vary between various tissues within the barley root apical meristem. This epigenetic turnover can be consider both along the longitudinal (from the root cap to elongation zone cells) and across the transversal (from the epidermis to the metaxylem cells) axis of the meristem.

## Discussion

To date there has been no clear evidence for differential levels of histone and DNA modification across root meristematic tissues. The results presented in this paper clearly indicate that levels of modifications with potential epigenetic effects do vary between RAM tissues. Differences were found in both the longitudinal axis, i.e. from the root cap, distal meristem, proximal meristem and elongation zone, and in the transverse axis, i.e. from the epidermis and the cortex to the vascular tissues.

Histone acetylation plays an important role during plant development. Long et al. [33] revealed that *AtGCN5*, one of the histone H3 acetylases, is required for root meristem activity. Mutants of *AtGCN5* show defects in root quiescent centre organization and root meristem differentiation [34]. Our results indicate that H4K5ac is present at a very high level in the vascular cylinder and, in contrast, at a very low level in epidermis. Hypothetically, distinct levels of this modification in particular tissues may be connected with vascular tissue differentiation. As histone acetylation is involved in gene activation, increased global levels of this modification can be expected in tissues which undergo differentiation. We also observed that the level of H4K5ac in the epidermis differs among various parts of the meristem (from almost undetectable in proximal meristem to much higher in the boundary between the proximal meristem and the elongation zone) and, additionally, between cells from the same part of the meristem. This observation can be attributed to epidermal cell differentiation into trichoblasts and atrichoblasts. It is known that histone acetylation and methylation are involved in the regulation of epidermal patterning of the gene *GLABRA2* (*GL2*), that encodes a homeodomain protein required for normal trichome development in Arabidopsis [35], [36]. Conversely, histone H4 acetylation of lysines 5 and 12 is deposition related (acetylated isoforms are incorporated into newly replicated chromatin), and appears to be a highly conserved phenomenon [37]. This may imply that the high level of this modification observed in particular tissues reflects replication. Replication-dependent H4K5ac was reported for in *Vicia faba*, where it was most abundant in the nuclei during or shortly after DNA replication [16].

There is no clear explanation for the presence of the H4K5ac immunosignals in the cytoplasm of the cortex and epidermis cells, but indirect evidence comes from studies in other eukaryote systems. It is known that diacetylation of nascent human histone H4 (H4K5ac) is completed prior to nucleosome assembly, hence H4 exists in the cytoplasm in a diacetylated form [38]. Another possible explanation comes from studies on yeast, where a model has been proposed in which histones are synthesized in the cytoplasm and then bound in their unmodified state by karyopherins (proteins which import histone H3 and H4 into the nucleus). The complexes subsequently bind histone acetyl transferase-B which promotes acetylation, which in turn may play a role in the release of the histones from karyopherins in the nucleus [39].

Histone H3 methylation at lysine 4 is a typical marker for euchromatin and plays a role in gene transcription. Our results indicate that this modification is present at the highest level in nuclei in the vascular cylinder; surprisingly, in other tissues the level of this modification is very low in nuclei, but high in the cytoplasm. Recently, Petruk et al. [40] revealed that in *Drosophila* embryos histone H3 trimethylated at lysine 4 is present during transcription but, surprisingly, is replaced by nonmethylated H3 following DNA replication. Methylated H3 is detected on DNA only in nuclei which are not in the S phase. These authors suggested that parental H3K4me3 is not transferred to original sites on nascent DNA but *de novo* methylation of H3 occurs only in the next G phase. Cytoplasmic immunosignals observed in our study may be the result of H3K4me2 displacement from nuclei and subsequent movement into the cytoplasm.

The phenomenon of high levels of H3K4me2 in the cytoplasm of root cap cells can possibly be explained by extensive DNA degradation in the nuclei of these cells, which we confirmed by the TUNEL test (data not shown). A similar observation was reported in protophloem cells, in which degradation of the nuclear DNA takes place during differentiation of this tissue [41]. It cannot be ruled out that the high content of H3K4me2 in the root cap cells might be involved in root cap development. As border cells (i.e. the cells produced by the root cap meristem that separate from the rest of the root upon reaching the periphery of the cap) detach from roots, a complex of proteins known as the root cap secretome is synthesized and exported from living cells into the matrix ensheathing the root tip [42]. Histone H4 and extracellular DNA were found among these proteins [43].

Histone H3 methylation at lysine 9 and DNA methylation at cytosine residues are considered to be landmarks for heterochromatin. Our results indicated that both 5mC and H3K9me2 were most abundant in nuclei of the epidermis from the boundary between proximal meristem and the elongation zone, as well as in the distal meristem, where the QC is located, and in the root cap cells. In the distal meristem, DNA methylation may involve cells of the QC. It was shown that the QC nuclei and nuclei of stem cells of cortex and endodermis were characterised by higher amounts of 5mc, whereas nuclei of the vascular tissue usually displayed a lower level of DNA methylation. Moreover, a high level of cytosine methylation was postulated to be an integral part of the QC identity [44]. The epidermis was another tissue where high levels of 5mC were found. Interestingly, it was predominant at the same root zone (i.e. the boundary between proximal meristem and the elongation zone) as the high level of H4K5ac. Based on these results we may speculate that these two modifications may play some significant role during epidermal cell development. Last but not least, differentiated levels of H3K9me2 in the epidermal cells (high in some cells but almost undetectable in others) may imply that this modification could be involved in the process of trichoblast and atrichoblast cell development. This hypothesis needs further experimental confirmation.

## Materials and Methods

### Material and slide preparation

Root apical meristems were taken from 3-day old barley seedlings (*Hordeum vulgare* L., 2n = 14, cv. Start), fixed in 4% formaldehyde in PBS and then placed in a vacuum desiccator for 2 hours. To remove the fixative, the material was washed in PBS for 30 minutes, dehydrated in a graded series of ethanol in PBS solution (30%, 50%, 70%, 90%) for 30 minutes each and 99.8% ethanol twice for 30 minutes. The embedding medium was prepared from polyethylene glycol 400 distearate and 1-hexadecanol (9/1 w/w) [45]. Embedding was done at 37°C in a graded wax/ethanol series (1/2, 1/1, 2/1 v/v) for 24 hours each, followed by one change of pure wax for another 24 hours. Meristems were then placed into embedding molds and left to polymerize overnight at room temperature. Root meristems were sectioned to 5 µm-thick tissue sections using a Leica RM 2145 microtome, placed on poly-L-lysine-coated slides and stretched by the addition of a small drop of water. Slides were allowed to dry overnight at room temperature. After de-embedding three times for 10 minutes in 99.8% ethanol, followed by rehydration in ethanol/PBS for 10 min each step (90%, 70%, 50%, 30% v/v, PBS only), the sections were used for immunostaining.

### Immunostaining

The immunostaining was carried out as previously described [46], [47]. Briefly, the following rabbit monoclonal and polyclonal antibodies against modified histones and DNA were used: anti-acetyl histone H4 at lysine 5 (1∶100; Millipore, Cat. no. 04-118), anti-dimethyl histone H3 at lysine 4 (1∶100 dilution in 1% BSA in PBS; Millipore, Cat. no. 07-030 and Cat. no. 07-790), anti-dimethyl histone H3 at lysine 9 (1∶100; Upstate, Cat. no. 05-768 and 07-212), anti-5-methyl-cytosine (1∶300, Abcam, Cat. no. ab73938). As secondary antibodies, Alexa Fluor 488 goat anti-rabbit IgG (Invitrogen, Molecular Probes Cat. no. A-11008) and Alexa Fluor 488 goat anti-mouse IgG (Invitrogen, Molecular Probes, Cat. no. A-11001) were applied.

### Image acquisition and processing

Images of meristem cross sections were registered using an Olympus FV1000 confocal system. The quantitative acquisition and analysis were performed using a high-content screening system (Scan∧R, Olympus) based on a wide-field microscope Olympus IX81 equipped with a CCD camera ORCA-ER (Hammamatsu Photonics), and an MT20 illumination system based on a Xenon-mercury lamp, 150W. The automated segmentation of nuclei was based on threshold values (a border value of pixels fluorescence intensity between the background and the object). The analysis was performed assuming following parameters of fluorescence intensities: ‘total’ (the sum of the pixel intensity value specific for the object) and ‘mean’ (the total intensity divided by the area of the object). The levels of epigenetic modifications were measured as an average value from total Alexa 488 fluorescence intensities (for detail see Figure S1 and S2). Image processing (including maximum intensity projections of optical ‘z’ sections) operations was done using an ImageJ 1.41 (Wayne Rasband, National Institutes of Health, USA) as described previously [46], [47].

## Supporting Information

Figure S1 **Nuclei gated into low, medium and high level of H4K5ac.** **A–C.** Histograms showing correlation of total fluorescence intensity of DAPI and Alexa 488 in nuclei with low (**A**), medium (**B**) and high (**C**) level of H4K5ac. **D–F.** Examples of nuclei galleries of low (**D**), medium (**E**) and high (**F**) level of H4K5ac. **G–I.** The tissue-specific localisation of nuclei with low (**G**), medium (**H**) and high (**I**) level of H4K5ac. Exemplary nuclei are marked with red circles. R1 – nuclei with low, R2 – nuclei with medium, and R3 – nuclei with high level of H4K5ac. Red (false colour) - DAPI staining, green - Alexa 488 (immunostaining of H4K5ac).
(TIF)

Figure S2 **Exemplary quantitative analysis of the H4K5ac level.** **A–D.** Histograms showing correlation of DNA level (total fluorescence intensity of DAPI) and H4K5ac level (total fluorescence intensity of Alexa 488) within nuclei gated into low (red dots), medium (green dots) and high (blue dots) level of H4K5ac (**A**). The histogram showing correlation of the number and different levels of DNA within nuclei gated into low (red dots), medium (green dots) and high (blue dots) level of H4K5ac (**B**). The histogram showing the correlation of the number and different levels of H4K5ac within nuclei gated into low (red dots), medium (green dots) and high (blue dots) level of H4K5ac (**C**). The histogram showing the correlation of the area of nuclei with different level of DNA content (**D**).
(TIF)
