# Supplementary figures and images for: Tissue-Specific Epigenetic Modifications in Root Apical Meristem Cells of Hordeum vulgare
Source: PLoS One. 2013 Jul 31;8(7):e69204. doi: 10.1371/journal.pone.0069204 (PMC3729647; doi:10.1371/journal.pone.0069204)

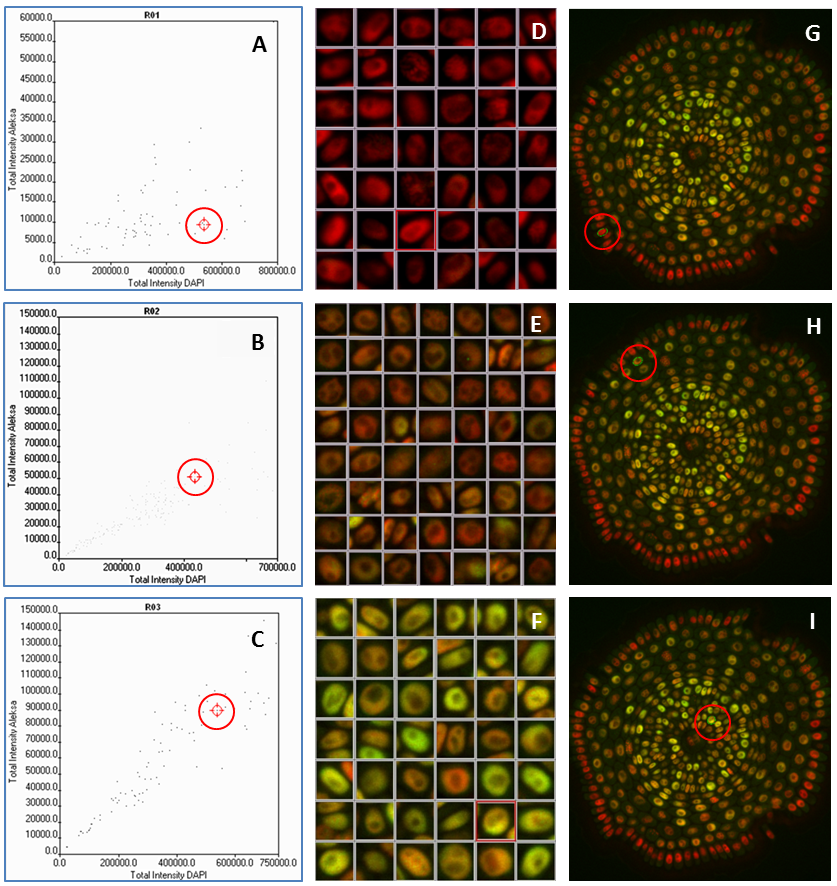

Supplement: Figure S1 — Nuclei gated into low, medium and high level of H4K5ac. A–C. Histograms showing correlation of total fluorescence intensity of DAPI and Alexa 488 in nuclei with low (A), medium (B) and high (C) level of H4K5ac. D–F. Examples of nuclei galleries of low (D), medium (E) and high (F) level of H4K5ac. G–I. The tissue-specific localisation of nuclei with low (G), medium (H) and high (I) level of H4K5ac. Exemplary nuclei are marked with red circles. R1 – nuclei with low, R2 – nuclei with medium, and R3 – nuclei with high level of H4K5ac. Red (false colour) - DAPI staining, green - Alexa 488 (immunostaining of H4K5ac). (TIF) [file pone.0069204.s001.tif]

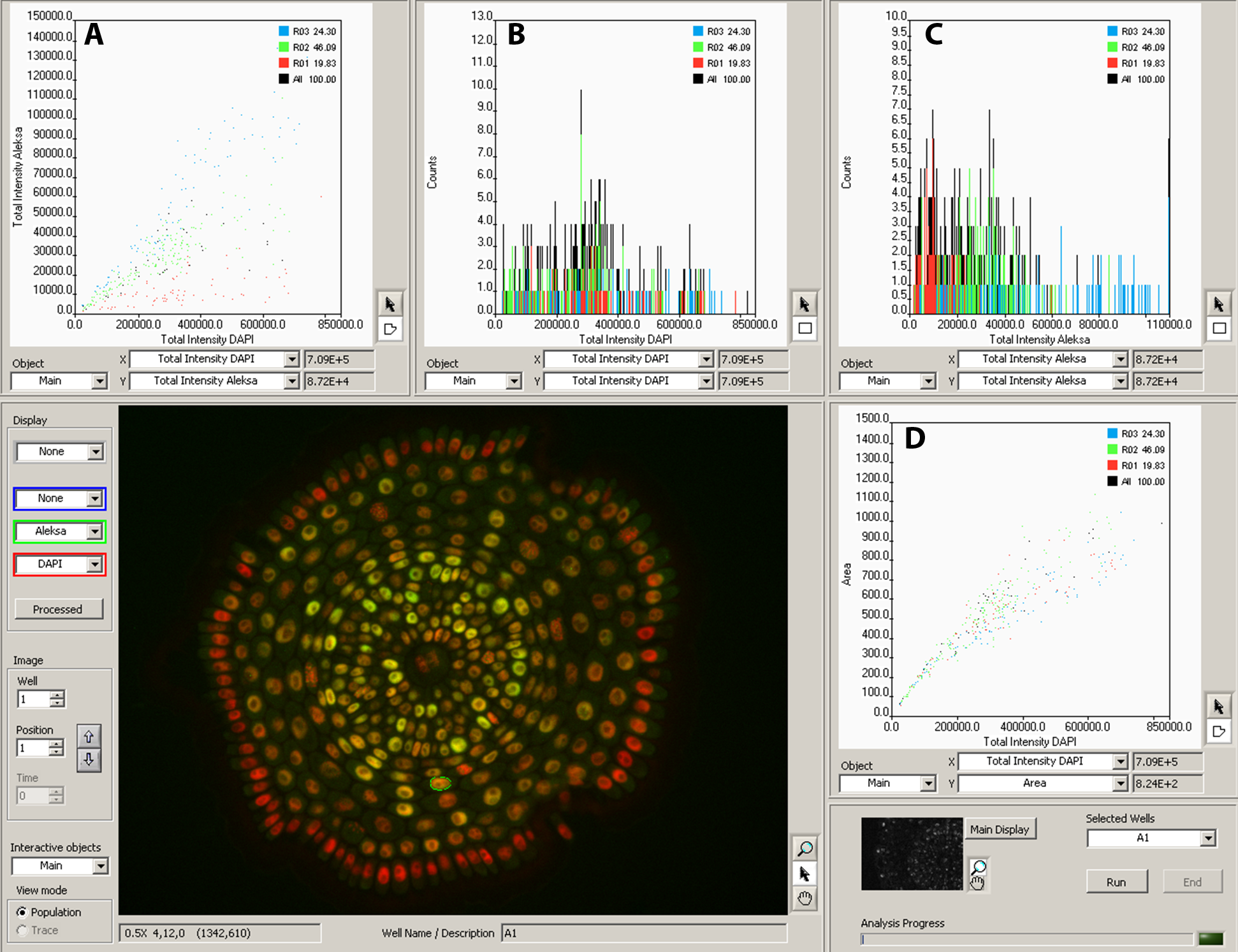

Supplement: Figure S2 — Exemplary quantitative analysis of the H4K5ac level. A–D. Histograms showing correlation of DNA level (total fluorescence intensity of DAPI) and H4K5ac level (total fluorescence intensity of Alexa 488) within nuclei gated into low (red dots), medium (green dots) and high (blue dots) level of H4K5ac (A). The histogram showing correlation of the number and different levels of DNA within nuclei gated into low (red dots), medium (green dots) and high (blue dots) level of H4K5ac (B). The histogram showing the correlation of the number and different levels of H4K5ac within nuclei gated into low (red dots), medium (green dots) and high (blue dots) level of H4K5ac (C). The histogram showing the correlation of the area of nuclei with different level of DNA content (D). (TIF) [file pone.0069204.s002.tif]
